# The Impact of the Successive Outbreaks of COVID-19, Vaccination, and Physical Activity on Mental Health in the Argentine Population: A Repeated Cross-Sectional Study

**DOI:** 10.7759/cureus.54932

**Published:** 2024-02-26

**Authors:** Alejo Ramiro Barbuzza, Fabricio Ballarini, Celina Goyeneche, Victoria Reppucci, Pedro Benedetti, Franco Moscato, Jorge H Medina, Cynthia Katche, Diego Moncada, Haydeé Viola

**Affiliations:** 1 Department of Life Sciences, Technological Institute of Buenos Aires (ITBA), Autonomous City of Buenos Aires, ARG; 2 Faculty of Medicine, Universidad de Buenos Aires-Consejo Nacional de Investigaciones Científicas y Técnicas (UBA/CONICET), Institute of Cell Biology and Neuroscience "Prof. E. De Robertis" (IBCN), Autonomous City of Buenos Aires, ARG; 3 Laboratory of Memory Neurophysiology, Institute of Cell Biology and Neuroscience "Prof. E. De Robertis" (IBCN), Autonomous City of Buenos Aires, ARG

**Keywords:** physical activity, vaccines, argentine population, depression, generalized anxiety disorder

## Abstract

Background and objectives

A controversy regarding the duration of generalized anxiety disorders (GAD) and depressive symptoms during the COVID-19 pandemic arose, stating that these symptoms last a short time, perhaps a few months, or that they are more persistent over time. After more than three years of the pandemic, this is still a question that requires an answer. The main goal of this work was to record the levels of self-perceived GAD and depression in the Argentine population at several time points during the pandemic to characterize whether they were transient or persisted over the successive waves of contagion. Furthermore, we studied the association between anti-COVID-19 vaccination and the high frequency of physical activity with GAD and depression levels to evaluate a possible protective role of these factors on mental health.

Methods

We used a descriptive and correlational research design. We carried out a repeated cross-sectional study performing seven online surveys (collection period: four to 15 days) at different time points in October 2020, May, August, October, and December 2021, and February and April 2022. The participants (24,308) were recruited through Instagram campaigns performed by renowned local scientific communicators and responded to the survey through Google Forms (Google, Mountain View, CA). Generalized anxiety was assessed using the Generalized Anxiety Disorder-7 (GAD-7). Depression was assessed using the Patient Health Questionnaire (PHQ-9). The respondents reported their symptoms using a four-point Likert scale, which led us to calculate the scores and also the prevalence (% of the population with moderate to severe symptoms) for GAD and depression and the frequency they performed physical activity per week. Data were statistically analyzed using the unpaired Mann-Whitney U-test, chi-squared, Spearman correlation, or Tukey's post hoc test after two-way ANOVA.

Results

Our results show that the highest prevalence for GAD and depression correspond to those of the second wave of infections (May 2021: 57.3% and 54.19%, respectively) and that the lower levels were reported by the end of the third wave (April 2022: 43.21% and 43.65%, respectively). Such levels were even lower than those reported during the first wave at the beginning of our study (October 2020: 45.94% and 48.92%, respectively). In other words, even though the third wave tripled the number of people infected with respect to the second one, its effects on mental health were attenuated. The increment in the vaccine doses inoculated between the last two waves of contagion was associated with a decrease in the GAD score (mean ± SEM: 10.75 ± 0.06 vs. 8.88 ± 0.13) and the depressive symptoms (mean ± SEM: 10.76 ± 0.07 vs. 9.23 ± 0.14). Throughout the entire study period, the fraction of the population that practiced physical activity three or more times per week was self-perceived with lower levels of GAD and depression than those who exercised less frequently.

Conclusions

Of the three waves of contagion that the Argentine population suffered, the highest rates of GAD and depression were recorded in the second wave, and these symptoms decreased over the months, even during the third wave, which presented the highest number of infections. Our results also suggest that the progress of the vaccination campaign and the practice of physical exercises with high frequency could play a protective role in the mental health of the population during COVID-19.

## Introduction

COVID-19 caused a plethora of negative effects on health, including an increase in the prevalence of depressive and anxiety disorders among the global population [1].

A controversy regarding the duration of generalized anxiety disorders (GAD) and depressive symptoms that emerged during the COVID-19 pandemic arose with the proposal of two opposing postulates. The first one states that, as has happened previously in other catastrophes or disaster exposures in modern societies, depressive and anxiety symptoms in the COVID-19 pandemic last a short time, perhaps a few months [2]. On the contrary, the second one states that the symptoms of altered mental health during the COVID-19 pandemic have lasted throughout 2020 [1]. After more than three years of the pandemic, whether the differences in the prevalence of mental health problems vary across the successive waves of infections, and how they did it, is still a question that requires an answer. Some longitudinal or repeated cross-sectional studies were carried out with this purpose, but their conclusions are diverse. In some cases, this could be due because those longitudinal studies covered a few months and/or contemplated different outbreaks of the pandemic [3-6]. The other works, covering long-term studies, showed either increased values of both the prevalence of GAD and depression compared to the beginning of the pandemic [7,8], or mixed results with ups and downs [9]. Probably, the country under study is a factor to consider in this analysis, because the impact of COVID-19 on mental health may be more significant in developing countries as social inequalities increase, vaccination is delayed, and lockdowns are extended.

Thus, the main goal of this work was to study the time course of GAD and depression records in Argentina throughout a repeated cross-sectional analysis within 19 months, covering three outbreaks of the COVID-19 pandemic. In parallel, we studied whether the levels of these parameters showed any relation to the practice of physical activity and whether it had a differential impact throughout the pandemic. Various studies have been carried out relating the frequency and intensity of physical activity with respect to its effectiveness in terms of mental health [10-13]. Here, our objective was to determine the distribution of the adult population that exercised with low or high weekly frequency and compare the self-reported scores of GAD and depression in these two groups of people to analyze whether physical activity could be associated with a protective function on mental health, even in the context of the chronic impact of this pandemic.

In this study, we report the moments with higher and lower GAD and depression scores and prevalence levels, identifying the more vulnerable groups concerning gender and age. We analyzed the degree of correlation of these parameters with the number of infections and deaths and we evaluated factors that could mitigate the negative consequences of these mental disorders, focusing on vaccination and physical activity.

## Materials and methods

Study overview

A multiple cross-sectional study was performed to survey the Argentinian adult population at seven different moments during the COVID-19 pandemic. This study was not designed to survey a specific population; randomly anyone over 18 years old with access to Instagram could participate in it.

Ethical considerations

This study was approved by the ethics council of the Life Sciences Department of the Instituto Tecnológico de Buenos Aires on August 10, 2020. All the procedures conducted in this study followed the ethical standards of the institutional and national research committees as well as those of the Helsinki Declaration of 1975, as revised in 2008. Consent was obtained or waived by all participants in this study.

Study criteria

We used a descriptive and correlational research design to associate the levels of GAD and depression with seven time points across the waves of contagion. We analyzed these levels considering the sex and age range of the population. Also, we carried out a correlational analysis between the GAD and depression scores, recorded in the different surveys, with their respective values of confirmed COVID-19 cases, number of deaths, and applied vaccine doses. Finally, we analyzed the association between the GAD and depression levels obtained in each survey with the frequency of physical activity reported by the respondents. All participants were recruited through Instagram campaigns performed by renowned local scientific communicators and responded to the survey through the platform Google Forms (Google, Mountain View, CA). We anonymized the data upon each survey analysis after evaluating the existence of duplicated responses within each survey. A minimum amount of participants answered more than one time the same survey (less than 0.1 %) and the duplicates were removed from the analysis. Since this is a cross-sectional study, we analyzed the sample of each survey independently, therefore we did not evaluate the existence of possible longitudinal responses.

For this study, the responses of all people between 18 and 50 years of age who inhabit the Argentine territory were considered. The population was divided into two groups: people between 18 and 30 years old and people between 31 and 50 years old. This division was performed taking as reference the parameters used by the National Institute for Statistics and Census (INDEC) to differentiate between young (15-29 years) and adults (30-60 years) within the economically active population [14-17]. Although both groups may share certain lifestyles and characteristics, there are differences between them: young people are studying or have low-responsibility jobs. In Argentina, a large percentage of them live with their parents (64%) and do not have children (90%) [14]. On the other hand, the majority of people between 30 and 60 years old are the economic support of the family [15-17].

Study procedure

Data were collected through Google Forms. Seven online surveys were performed in October 2020 (from 22nd October 2020 to 7th November 2020, n = 2647, 78.54% women), May 2021 (from 5th May 2021 to 10th May 2021, n = 7326, 82.34% women), August 2021 (from 24th August 2021 to 31st August 2021, n = 4560, 87.48% women), October 2021 (from 7th October 2021 to 14th October 2021, n = 5013, 86.35% women), December 2021 (from 20th December 2021 to 24th December 2021, n = 1731, 87.92% women), February 2022 (from 31st January 2022 to 7th February 2022, n = 1418, 90.06% women), and in April 2022 (from 1st April 2022 to 4th April 2022, n = 1613, 91.88% women). Thus, considering these seven surveys, the total sample size of our study was 24,308 people between 18 and 50 years old. Participants responded to the surveys voluntarily without any direct contact or encouragement to do it.

In each survey, the participants informed their gender (men, women, and others), age (we only included people aged 18-50 years), and the province of residence, they completed the Generalized Anxiety Disorder Scale (GAD-7) and the Patient Health Questionnaire (PHQ-9) tests, and reported the weekly frequency they performed physical activity per week (zero up to seven). Although there was no time limit, respondents did not take more than 10 minutes. Only fully answered surveys were considered for the analysis.

Assessments

The GAD-7 [18,19] was used to measure generalized anxiety as previously described [13]. This mental health instrument gathers information about generalized anxiety symptoms of the two weeks previous to the questionnaire. Respondents report their symptoms using a four-point Likert rating scale ranging from 0 (not at all) to 3 (almost every day) along seven questions, therefore the total score ranges from 0 to 21. Scores of 0-4 are thought to represent minimal anxiety, 5-9 represent mild anxiety, 10-14 represent moderate anxiety, and 15-21 represent severe anxiety [18].

The PHQ-9 [20] was used to measure depression. The PHQ-9 resulted in a reliable and widely validated measure of depressive symptoms [21]. Each respondent must answer nine questions that describe depression symptoms, considering the last two weeks. Each question can be answered with a four-point Likert rating scale ranging from 0 (not at all) to 3 (almost every day) along nine questions, thus the total score ranges from 0 to 27. Scores of 0-4 suggest minimal depression, 5-9 suggest mild depression, 10-14 suggest moderate depression, 15-19 suggest moderately severe depression, and 20-27 suggest severe depression [20].

Sample size calculation

Considering the proportion of people between 18 and 50 years old living in Argentina, at least 379 people are required to have a confidence level of 95% with a 5% margin error. Because we surveyed people using a convenient online method, we decided to maximize the sample size of each survey by including all participants within that age range who responded to it within the data collection period.

Statistical analysis

For continuous variables (GAD score from GAD-7 and depression score from PHQ-9), descriptive statistics were calculated and expressed as means with standard error of the mean (SEM). For non-continuous variables, descriptive statistics were calculated and expressed as counts and percentages (%). Samples that did not follow a normal distribution or the homoscedasticity requirements of parametric tests were analyzed using non-parametric tests. Assuming a normal distribution of the means due to the large sample size, we calculated all the non-parametric statistics using the equivalent parametric test, and no differences between tests were found. The differences were considered significant when p < 0.05 (α = 0.05). The statistical analysis was performed using GraphPad Prism® 8.0.1 software (GraphPad Software, San Diego, CA). We used Spearman’s rank correlation coefficient (rs) to calculate the relation between GAD and depression scores. Data about new confirmed COVID-19 cases per 1M, new confirmed COVID-19 deaths per 1M accumulated in the 14 days before each survey, and vaccine doses per 100 people accumulated until 14 days before each survey were extracted from Our World in Data.

## Results

In total, 24,308 participants between 18 and 50 years old responded to an online survey between October 2020 and April 2022. The demographic distributions of the respondents in each survey during the COVID-19 pandemic in Argentina are shown in Table 1.

**Table 1:** Demographic distribution of the respondents in each survey during the COVID-19 pandemic in Argentina. The table shows for each survey (1st column) and each gender (woman, men) and age group (18-30; 31-50) (2nd column), the total number of respondents (N) and their corresponding % of the sample (3rd column).

Surveys	Group	N (%)
October 2020	Women 18-30	964 (36.41%)
	Women 31-50	1115 (42.13%)
	Men 18-30	291 (10.99%)
	Men 31-50	277 (10.46%)
May 2021	Women 18-30	2534 (34.59%)
	Women 31-50	3498 (47.75%)
	Men 18-30	676 (9.22%)
	Men 31-50	618 (8.44%)
August 2021	Women 18-30	1312 (28.77%)
	Women 31-50	2677 (58.71%)
	Men 18-30	255 (5.59%)
	Men 31-50	316 (6.93%)
October 2021	Women 18-30	1502 (29.96%)
	Women 31-50	2827 (56.39%)
	Men 18-30	294 (5.86%)
	Men 31-50	390 (7.78%)
December 2021	Women 18-30	413 (23.86%)
	Women 31-50	1109 (64.06%)
	Men 18-30	91 (5.26%)
	Men 31-50	118 (6.82%)
February 2022	Women 18-30	347 (24.47%)
	Women 31-50	930 (65.59%)
	Men 18-30	55 (3.88%)
	Men 31-50	86 (6.07%)
April 2022	Women 18-30	342 (21.20%)
	Women 31-50	1140 (70.68%)
	Men 18-30	44 (2.73%)
	Men 31-50	87 (5.39%)

**Figure 1:**
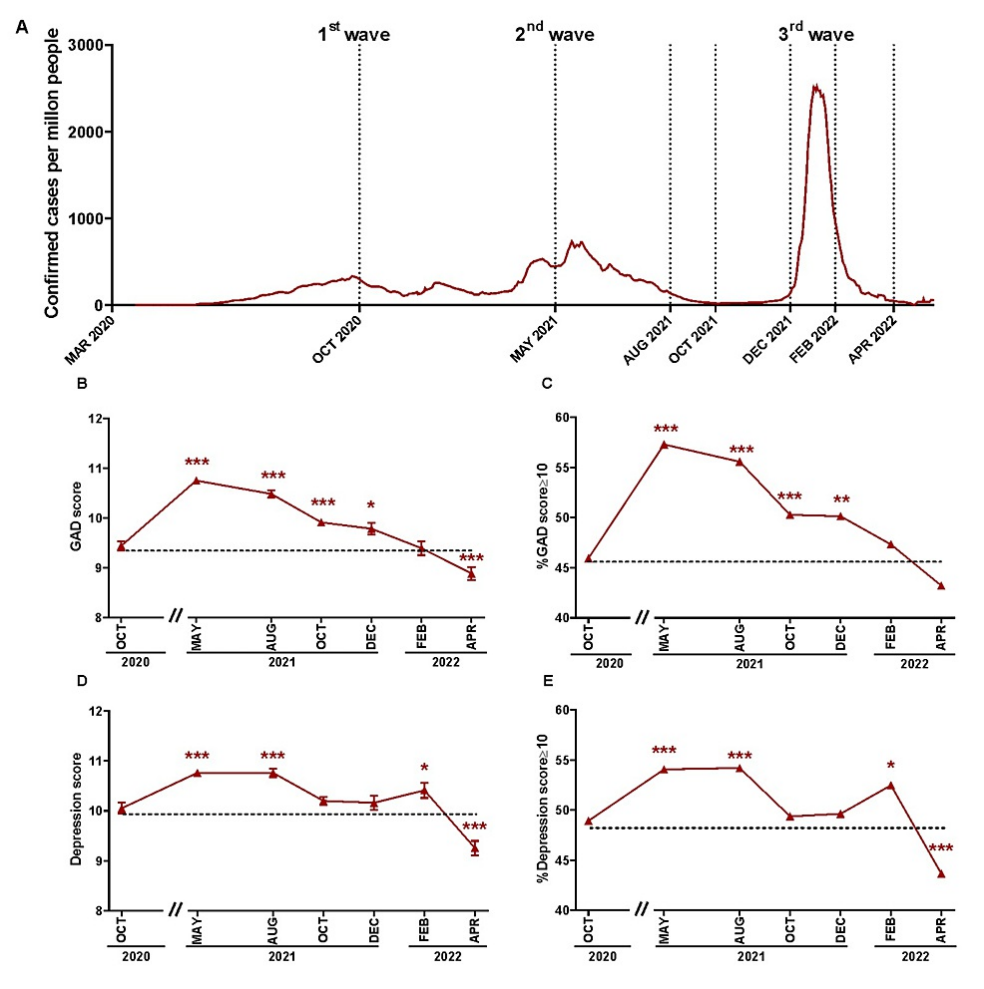
Changes in the Argentine population's (18-50 years) GAD and depression scores during the COVID-19 pandemic. The figure shows the mean ± SEM of generalized anxiety disorder (GAD) and depression scores or the percentage of people aged between 18 and 50 with GAD and depression scores ≥10 between October 2020 and April 2022. (A) Daily confirmed COVID-19 cases per million people from 1st March 2020 to 10th May 2022. Dotted lines indicate the midpoint corresponding to the period of time covered by each survey. Data extracted from Our World in Data. (B) GAD scores during the COVID-19 pandemic in comparison to the first data collection (October 2020, dotted line). Unpaired Mann-Whitney U-test (*** p < 0.001; * p < 0.05). (C) Prevalence of moderate to severe GAD during the COVID-19 pandemic in comparison to the first data collection (October 2020, dotted line). Chi-squared (*** p < 0.001; ** p < 0.01). (D) Depression score during the COVID-19 pandemic to first data collection (October 2020, dotted line). Unpaired Mann-Whitney U-test (*** p < 0.001; * p < 0.05). (E) Prevalence of moderate to severe depression during the COVID-19 pandemic in comparison to October 2020. Chi-squared (*** p < 0.001; * p < 0.05).

Figure 1A shows the infections per million people in Argentina between October 2020 and April 2022 and the dotted lines indicate when the surveys were conducted. The first one was carried out in October 2020, at the peak of the first outbreak. The second one was carried out in May 2021, matching the second peak of infections. The following two surveys were in August and October 2021 when this outbreak was in downfall and remission, respectively. Then, we carried out the fifth survey in December 2021, at the beginning of the high peak of infections due to the Omicron variant. The last two surveys were performed in February and April 2022 when this third peak was in downfall and remission, respectively. The mean reported score for GAD in October 2020 was 9.43 (Figure 1B), and this value will be used to compare with the records obtained in subsequent surveys. The highest records of GAD were reported in May and August 2021, reaching a maximum score of 10.75 (Figure 1B, p < 0.001). From October 2021 onwards, people gradually reported lower GAD scores. In April 2022, we registered lower GAD levels than in the first wave of infections (Figure 1B, p < 0.001). This behavior was matched by the high prevalence of moderate to severe GAD, which reached more than 55% of the population surveyed in May and August 2021 (Figure 1C, p < 0.001 vs. October 2020), and progressively decreased up to April 2022.

As for the depression, the mean score during October 2020 was 10 (Figure 1D). Then, at the second outbreak, higher levels were reported compared to the first peak of contagions (Figure 1D, p < 0.001) while in April 2022, the score decreased to 9.25 (Figure 1D, p < 0.001). In turn, the prevalence of moderate to severe depression symptoms was higher in May 2021, August 2021, and February 2022 than in the first outbreak (Figure 1E, p < 0.001, p < 0.001, and p = 0.03, respectively), and then decreased by April 2022 (Figure 1E, p < 0.001). In summary, the symptoms of GAD and depression were higher during the second peak of infections compared to the first one. However, this response was not sustained at the population level during the third peak of infections, and even lower values were recorded at the end of the third peak in comparison to the beginning of our study.

**Figure 2:**
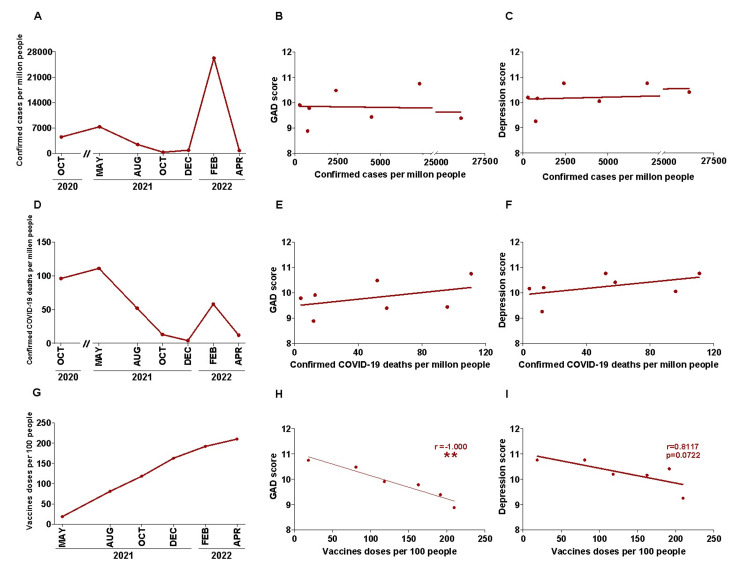
Analysis of the relation between generalized anxiety disorder (GAD) and depression scores with confirmed COVID-19 cases, deaths, and vaccination during the COVID-19 pandemic. (A) Number of confirmed COVID-19 cases per million people^‡^ and its correlation with (B) GAD score (Spearman correlation, r = 0.07143, p > 0.05) or (C) depression score (r = 0.4685; p > 0.05). (D) The mean number of confirmed COVID-19 deaths per million people^‡^ and its correlation with (E) GAD score (r = 0.3571; p > 0.05) or (F) depression score (r = 0.4865; p > 0.05). (G) Vaccine doses per 100 people^#^ and its correlation with (H) GAD score (r = -1.000; ** p = 0.002) and (I) depression score (r = -0.8117; p > 0.05). ^‡^ Accumulated in the 14 days before each survey. ^#^ Accumulated until 14 days before each survey.

The fact that mental health symptoms were not fully related to the number of COVID-19 infections can be clearly observed in Figures 2B, 2C. We evaluated the relationship between GAD and depression scores to the amount of new COVID-19-positive cases reported. As the questionnaires reveal the self-perception of symptoms related to these emotional states through the two weeks prior to the day of the survey, we calculated the cases of contagion that occurred in that period in Argentina for each of the surveys and made a correlation with the corresponding scores (Figure 2A). Neither GAD (Figure 2B, r: 0.071) nor depression scores (Figure 2C, r: 0.468) correlated to the number of new cases within the period of analysis. In addition, Figures 2E, 2F show the regressions between the GAD (Figure 2E, r: 0.357) and depression scores (Figure 2F, r: 0.486) with respect to the number of new deaths per million people in the period of 14 days prior to the surveys (Figure 2D). Finally, we found an inverse correlation between the GAD (Figure 2H, r: -1.00, p = 0.002) and depression scores (Figure 2I, r: -0.811, p = 0.07) with respect to the doses of vaccines per 100 people accumulated until 14 days before each survey (Figure 2G); the administration started in May 2021, reaching the 82% of inoculated in the Argentine population by the time of the last survey of our study.

**Figure 3:**
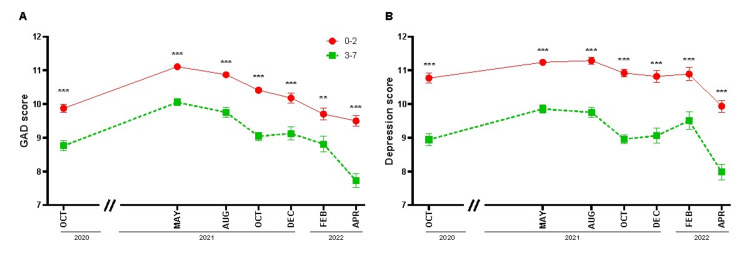
Practicing physical activity more than two days per week is associated with lower generalized anxiety disorder (GAD) and depression throughout the pandemic. The figure shows the mean + SEM of GAD scores and depression scores between October 2020 and April 2022. For the two panels, the red line represents the group of people who practiced physical activity with low frequency (zero to two, days per week), and the green line represents those who did it with high frequency (three to seven, days per week). (A) GAD score in each survey for both physical activity groups during the COVID-19 pandemic. Unpaired Mann-Whitney U-test (*** p < 0.001; ** p < 0.01). (B) Depression score for both physical activity groups during the COVID-19 pandemic. Unpaired Mann-Whitney U-test (*** p < 0.001).

Since performing physical activity was associated with lower levels of GAD and depression [12], the survey also inquired the participants about the frequency they performed exercise. We clustered them into two groups, those who exercised up to two days per week (low frequency) and those who did it three days or more (high frequency). We observed that in all surveys, the high frequency of physical activity was associated with lower GAD (Figure 3A, p < 0.001 for all surveys except February 2022, p = 0.002) and depression levels (Figure 3B, p < 0.001). In addition, the reports provided throughout the entire pandemic showed that nearly 40% of people engaged in high physical activity in October 2020, but this value remained around 35% in the rest of the surveys, suggesting a decrease in this healthy practice in the pandemic (Table 2).

**Table 2:** Descriptive data and statistics corresponding to Figure 3. Percentage of people performing low (zero to two days per week) and high (three to seven days per week) physical activity frequency in each survey. Fisher's exact test analysis.

Survey	% people performing physical activity 0-2 days per week	% people performing physical activity 3-7 days per week	P-value vs. October 2020
October 2020	60.22	39.78	-
May 2021	65.38	34.62	<0.001
August 2021	65.5	34.5	<0.001
October 2021	63.1	36.9	0.0118
December 2021	62.39	37.61	0.1395
February 2022	64.88	35.12	0.0031
April 2022	65.16	34.84	0.0011

**Figure 4:**
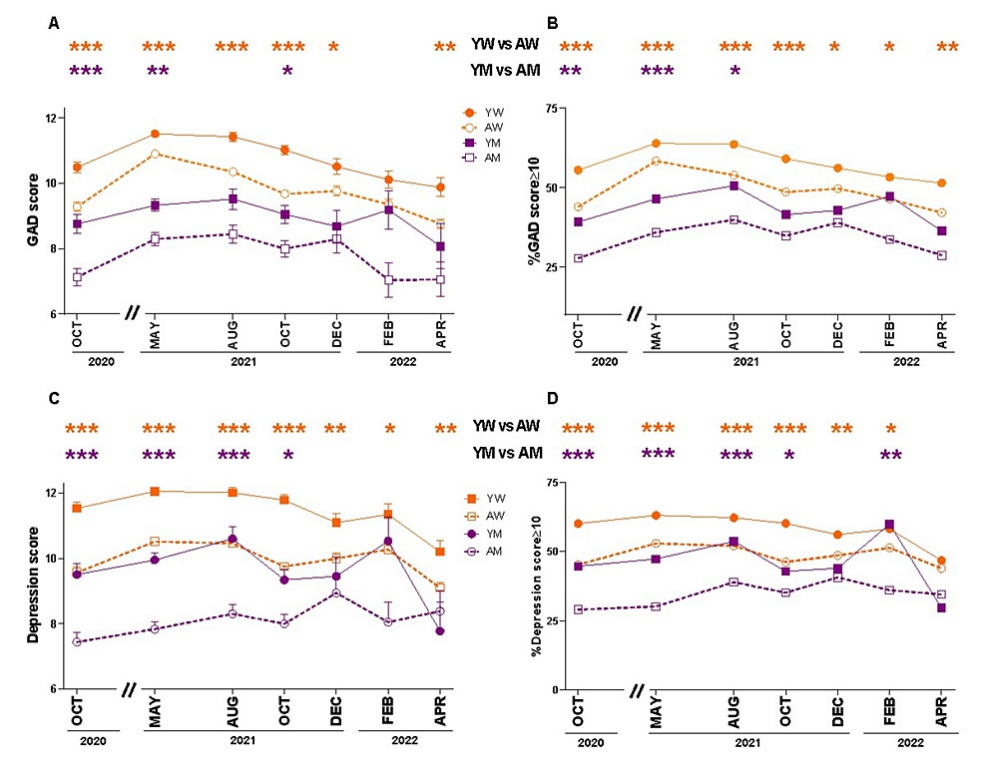
Changes in the Argentine population's generalized anxiety disorder (GAD) and depression during the COVID-19 pandemic classified by gender and age group. The figure shows the mean ± SEM of GAD and depression scores and the percentage of people with GAD and depression scores ≥10 for women and men aged between 18 and 30 years or 31 and 50 years (YW, AW, YM, and AM, respectively) between October 2020 and April 2022. (A) GAD scores in each survey for both genders and age groups during the COVID-19 pandemic. Tukey's post hoc test after two-way ANOVA (*** p < 0.001, ** p < 0.01, and * p < 0.05). (B) Prevalence of moderate to severe GAD in each survey for both genders and age groups during the COVID-19 pandemic. Chi-squared (*** p < 0.001, ** p < 0.01, and * p < 0.05). (C) Depression scores in each survey for both genders and age groups during the COVID-19 pandemic. Tukey's post-hoc test after two-way ANOVA (*** p < 0.001, ** p < 0.01, and * p < 0.05). (D) Prevalence of moderate to severe depression in each survey for both genders and age groups during the COVID-19 pandemic. Tukey's post-hoc test after two-way ANOVA (*** p < 0.001, ** p < 0.01, and * p < 0.05). For all panels: YW (full circles), AW (empty circles), YM (full squares), and AM (empty squares). YW: young women; AW: adult women; YM: young men; AM: adult men.

Then, we analyzed the scores and prevalence of GAD and depression according to the gender and age of the surveyed population (Figure 4). We observed a gender effect in which women evidenced higher levels than men along the seven surveys (Table 3, p < 0.05 - 0.001). In addition, we analyzed possible age effects inside each gender. We observed that the young women (YW, 18-30 years old) always reported higher scores and prevalence of GAD than those of adult women (AW, 31-50 years old) (Figures 4A, 4B, p < 0.05 - 0.001). Moreover, we observed that only at the first two infection outbreaks, the young men (YM, 18-30 years old) reported higher scores and prevalence of GAD than those of adult men (AM, 31-50 years old) (Figures 4A, 4B, p < 0.05 - 0.001). With respect to depression, the YW had higher scores and prevalence than AW throughout the entire pandemic (Figures 4C, 3D, p < 0.05 - 0.001). The YM group reported higher depression scores and prevalence levels than AM until the end of the second peak of infections (Figures 4C, 3D, p < 0.05 - 0.001). Thereafter, they evidenced similar values except in February 2022 where an increase in prevalence was observed in the YM group (Figure 4D, p = 0.005). In summary, the levels of GAD and depression throughout the pandemic were higher in women than in men and, in each gender, the youngest age group was the most affected.

**Table 3:** Gender effect corresponding to Figure 4. The table shows the p-value obtained after the two-way ANOVA or chi-square test when comparing the group of women vs. men.

	P-value, Gender effect. Two-way ANOVA (Figure 4A)	P-value, Gender effect. Chi-square (Figure 4B)	P-value, Gender effect. Two-way ANOVA (Figure 4C)	P-value, gender effect. Chi-square (Figure 4D)
October 2020	<0.001	<0.001	<0.001	<0.001
May 2021	<0.001	<0.001	<0.001	<0.001
August 2021	<0.001	<0.001	<0.001	<0.001
October 2021	<0.001	<0.001	<0.001	<0.001
December 2021	<0.001	0.0035	0.0024	0.0204
February 2022	<0.001	0.0372	0.0042	0.0761
April 2022	<0.001	0.0041	0.0049	0.0092

## Discussion

Until the report of this research, Argentina went through three waves of COVID-19 infections. During the first outbreak, no vaccines for this virus were yet developed in the world. The second wave of COVID-19 infections took place in the first half of 2021, with its peak of infections during May and June. The third wave, which was caused by the Omicron variant, occurred in January to February 2022 and was the most important in terms of the number of infections, but the least lethal, because around 80% of the local population was vaccinated. We can conclude that the highest prevalence levels of GAD and depression correspond to those of the second wave of infections (57.3% and 54.19%, respectively) and that the lower levels were reported by the end of the third wave (43.21% and 43.65%, respectively). Such levels were even lower than those reported during the first wave at the beginning of our study (45.94% and 48.92%, respectively). In other words, even though the third wave tripled the number of people infected with respect to the second one, its effects on mental health were attenuated.

We have reported that in May 2021, the prevalence of GAD and depression reached values ​​of 57.25% and 54.39%, respectively, considering a population restricted to the metropolitan area of ​​Buenos Aires [13]. Here, ​​we found similar values to those ones, even considering a population spread throughout the entire country. Undoubtedly, the records obtained during the second wave of infections in Argentina exceeded those obtained during the first one, reported by different research groups [8,22-24]. However, until now, no other work analyzed the impact of the third wave on mental health in Argentina. In that sense, our study indicates that the effects of COVID-19 on GAD and depression, at the population level, reached a maximum during the second wave, which was not sustained in the subsequent wave of contagion.

Considering that the cases of infections could be one of the factors affecting mental health, in our present work, we decided to analyze the relation between the cases of contagion with the levels of GAD and depression. We observed that in the second wave, despite the decrease in infections observed in August 2021, GAD and depression scores were still very high. Similar results were observed by Liao et al., who found that the highest rates of anxiety and depression were detected after the peak of the newly diagnosed cases per day [25]. These results show that self-reported changes in GAD and depression do not always reflect the situation regarding the number of infections. When these mental symptoms were studied in relation to the number of deaths, we observed a minor positive but non-significant correlation. However, the symptoms of GAD and depression in our country correlated inversely with the accumulation of vaccine doses applied. These data are in agreement with our previous observations, showing that vaccination is associated with a decrease in depressive symptoms during the second wave of the COVID-19 pandemic in the Argentine population [13]. Also, compared with unvaccinated populations, vaccinated populations had a significantly lower prevalence rate of depression and anxiety in a cross-sectional study performed in Bangladesh during the second semester of 2021 [26]. This fact supports that the vaccination campaign would provide a feeling of sanitary well-being [27] along the waves of contagion and represent a healthy behavior that benefits the mental health of the population.

Our results also suggest that another healthy behavior is the regular practice of physical activity. Throughout the 19 months of the pandemic and in the seven surveys we carried out, it was always observed that the group of people performing physical activity with high frequency was associated with lower levels of GAD and depression.

Then, in the present study, we also analyzed whether the gender factor influenced the score or the prevalence of GAD and depression. The results obtained throughout the three waves of contagion show that the self-reported levels of GAD and depression in the group of women exceeded in almost all cases the levels reported by the group of men. Similar results were obtained by Del-Valle et al. [28] during the first 13 months of the pandemic, showing progressive increases in anxiety and depression symptoms in the general Argentine population with significant effects on female gender, younger age, and lower income. The longitudinal study conducted on a population of Polish university students also found a gender effect along the three waves [9]. A short longitudinal study performed in England from March to July 2020 found that being a woman and younger were risk factors for higher levels of anxiety and depression [5]. Also, the COVID-19 Mental Disorders Collaborators performed a meta-analysis in 2020 and showed that women were more affected than men [1].

Another variable usually found as a risk factor for psychological problems is the age of the surveyed population. Women of younger age were one of the groups at higher risk for increased anxiety and depression symptoms in Cyprus, the UK, and Korea [5,29,30]. A similar result was obtained in our research, being this group the most affected by the pandemic. Also, within men, the young subgroup exhibited higher levels of GAD and depression symptoms. Age is a risk factor for mental distress in the Argentine population and in the world [7,23].

Our work bases its strength on the large sample size and its relative representability for recruiting the participants of the general population. However, similarly to other cross-sectional online surveys, this work has limitations. This type of study has implicitly a self-selection bias. For instance, in the present study, the number of women who answered the survey is almost four times higher than the number of men. Another limitation is reflected in the chosen design. The cross-sectional design does not necessarily apply to a particular individual. Instead, it reflects the benefits and risks for different groups in a given population. This limitation could mitigate the impact of the conclusions one can reach using the GAD-7 and PHQ-9 questionnaires, which are well-established tools in clinical practice and research. An additional limitation is that most respondents are young to middle-aged people. Finally, the number of people who participated in the different surveys was variable (see Table 1).

## Conclusions

The effects of the COVID-19 pandemic on the mental health of the Argentine population were not sustained along the three outbreaks. A high increase in GAD and depression symptoms accompanied the second wave of contagion and a sharp decline in these levels was parallel to the third wave, although it was the one with the highest number of infections. The young adult women group was the most vulnerable. Our results show that vaccination and frequent physical activity practices could contribute to reducing the risk of GAD and depression, highlighting the need to develop strategies to motivate these behaviors.
